# 1-Cyano­methyl-1,4-diazo­niabicyclo­[2.2.2]octane tetra­chloridocadmate(II)

**DOI:** 10.1107/S1600536812017801

**Published:** 2012-04-28

**Authors:** Yi Zhang, Bo Han Zhu

**Affiliations:** a Ordered Matter Science Research Center, Southeast University, Nanjing 211189, People’s Republic of China

## Abstract

In the title salt, (C_8_H_15_N_3_)[CdCl_4_], four Cl atoms coordinate the Cd^II^ atom in a slightly distorted tetra­hedral geometry. In the crystal, each [CdCl_4_]^2−^ anion is connected to the 1-cyano­methyl-1,4-diazo­niabicyclo­[2.2.2]octane dications by N—H⋯Cl hydrogen bonds, forming chains parallel to [001]. C—H⋯Cl inter­actions also occur.

## Related literature

For the use of 1,4-diaza­bicyclo­[2.2.2]octane (DABCO) and its derivatives, see: Basaviah *et al.* (2003 ▶); Zhang, Cheng *et al.* (2009 ▶). For ferroelectric properties of DABCO derivatives, see: Zhang, Ye *et al.* (2009 ▶, 2010 ▶). For related structures, see: Cai (2010 ▶); Wei (2010 ▶). For the isotypic cobaltate(II) analogue, see: Zhang & Zhu (2012 ▶). 
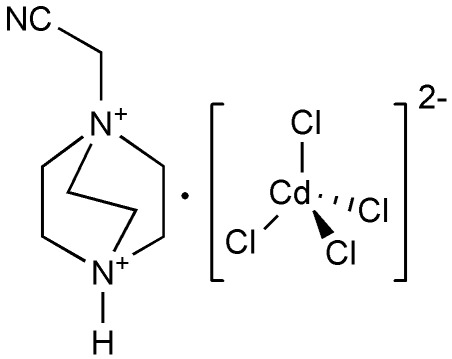


## Experimental

### 

#### Crystal data

(C_8_H_15_N_3_)[CdCl_4_]
*M* *_r_* = 407.43
Monoclinic, 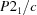

*a* = 8.3747 (17) Å
*b* = 13.772 (3) Å
*c* = 12.153 (2) Å
β = 93.89 (3)°
*V* = 1398.4 (5) Å^3^
*Z* = 4
Mo *K*α radiation
μ = 2.30 mm^−1^
*T* = 298 K
0.36 × 0.32 × 0.28 mm

#### Data collection

Rigaku SCXmini diffractometer
Absorption correction: multi-scan (*CrystalClear*; Rigaku, 2005 ▶) *T*_min_ = 0.441, *T*_max_ = 0.525
14246 measured reflections
3200 independent reflections
2899 reflections with *I* > 2σ(*I*)
*R*_int_ = 0.038

#### Refinement

*R*[*F* ^2^ > 2σ(*F* ^2^)] = 0.026
*wR*(*F* ^2^) = 0.059
*S* = 1.15
3200 reflections
150 parameters
H atoms treated by a mixture of independent and constrained refinement
Δρ_max_ = 0.46 e Å^−3^
Δρ_min_ = −0.48 e Å^−3^

### 

Data collection: *CrystalClear* (Rigaku, 2005 ▶); cell refinement: *CrystalClear*; data reduction: *CrystalClear*; program(s) used to solve structure: *SHELXS97* (Sheldrick, 2008 ▶); program(s) used to refine structure: *SHELXL97* (Sheldrick, 2008 ▶); molecular graphics: *SHELXTL* (Sheldrick, 2008 ▶); software used to prepare material for publication: *SHELXL97*.

## Supplementary Material

Crystal structure: contains datablock(s) I, global. DOI: 10.1107/S1600536812017801/pv2531sup1.cif

Structure factors: contains datablock(s) I. DOI: 10.1107/S1600536812017801/pv2531Isup2.hkl

Additional supplementary materials:  crystallographic information; 3D view; checkCIF report

## Figures and Tables

**Table 1:** Hydrogen-bond geometry (Å, °)

*D*—H⋯*A*	*D*—H	H⋯*A*	*D*⋯*A*	*D*—H⋯*A*
N3—H1⋯Cl2^i^	0.79 (3)	2.54 (3)	3.193 (2)	141 (3)
N3—H1⋯Cl3^ii^	0.79 (3)	2.76 (3)	3.285 (2)	126 (3)
C1—H1*A*⋯Cl3^iii^	0.97	2.70	3.507 (3)	141 (2)
C3—H3*B*⋯Cl4^iv^	0.97	2.67	3.599 (3)	160 (2)
C4—H4*A*⋯Cl1^i^	0.97	2.81	3.704 (3)	153 (2)
C7—H7*A*⋯Cl2^iv^	0.97	2.61	3.514 (3)	155 (2)
C7—H7*B*⋯Cl4^v^	0.97	2.79	3.489 (3)	129 (2)

Symmetry codes: (i) 
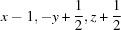
; (ii) 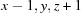; (iii) 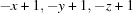; (iv) 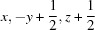; (v) 
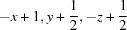
.

**Fig. 1.**
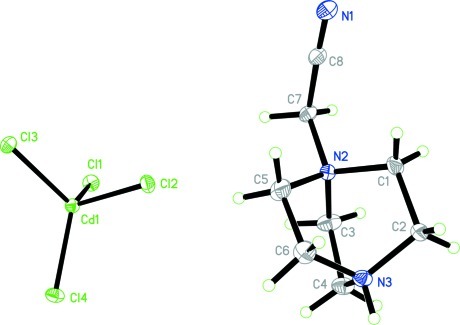
The molecular structure of the title compound with the atom numbering scheme. Displacement ellipsoids are drawn at the 30% probability level. H atoms are presented as small spheres of arbitrary radius.

**Fig. 2.**
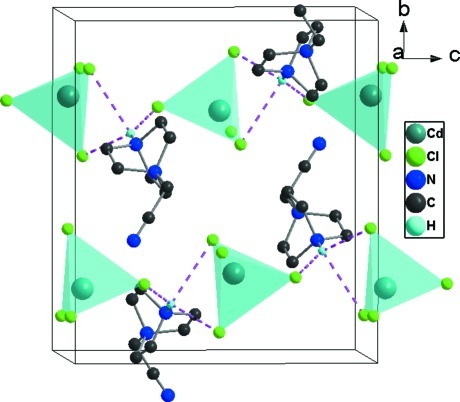
A view of the N—H···Cl hydrogen bonds (dotted lines) in the crystal structure of the title compound. H atoms non-participating in hydrogen-bonding were omitted for clarity.

## supplementary crystallographic information

### Comment

1,4-Diazabicyclo[2.2.2]octane (DABCO) is used as a effective organocatalyst for a large number of reactions because of its nucleophilicity (Basaviah *et al.*, 2003) and some of it's derivatives are ferroelectrics (Zhang, Cheng *et al.*, 2009). As part of a systematic investigation of dielectric-ferroelectric materials (Zhang, Ye *et al.*, 2009; 2010), we report the crystal structure of the title compound in this article.

The asymmetric unit of the title compound is composed of cationic (C_8_H_15_N_3_)^2+^ and anionic (CdCl_4_)^2-^ ions (Fig. 1). The Cd atoms are coordinated by four Cl atoms with very similar distances in the range of 2.2749 (12) to 2.2910 (12) Å. The Cl—Cd—Cl bond angles are between 103.21 (4) and 113.85 (5) ° which shows that the coordination polyhedron can be described as a slightly distorted tetrahedron. The ammonium groups of the organic cations are engaged in bifurcated hydrogen bonds to chlorine atoms of two (CdCl_4_)^2-^ anions. These weak N—H···Cl interactions cause the formation of a one-dimensional chain along the [0 0 1] (Fig. 2).

The crystal structures of a few related DABCO derivatives have been reported earlier (Cai, 2010; Wei, 2010).

### Experimental

Chloroacetonitrile (0.1 mol, 7.55 g) was added to a CH_3_CN (25 ml) solution of 1,4-diaza-bicyclo[2.2.2]octane (DABCO) (0.1 mol, 11.2 g) with stirring for 1 h at room temperature. 1-(Cyanomethyl)-4-aza-1-azonia-bicyclo[2.2.2]octane chloride quickly formed as white solid was filtered, washed with acetonitrile and dried (yield: 80%). CdCl_2_.2.5H_2_O (0.01 mol, 2.28 g) and 1 g 36% HCl were dissolved in H_2_O (20 ml) and 1-(cyanomethyl)-4-aza-1-azonia-bicyclo[2.2.2]octane chloride (0.01 mol, 1.875 g) in H_2_O (20 ml) was added. The resulting solution was stirred until a clear solution was obtained. After slow evaporation of the solvent, colourless needle crystals of the title compound suitable for X-ray analysis were obtained in about 60% yield. The title compound has no dielectric disuniform from 80 K to 373 K, (m.p. > 373 K).

### Refinement

The C-bound H atoms were positioned geometrically and refined using a riding model with C—H = 0.97 Å and *U*_iso_(H) = 1.2*U*_eq_(C). The H1 bonded to N3 was located from a difference Fourier map and freely refined.

### Crystal data

**Table:**

(C_8_H_15_N_3_)[CdCl_4_]	*F*(000) = 800
*M**_r_* = 407.43	*D*_x_ = 1.935 Mg m^−^^3^
Monoclinic, *P*2_1_/*c*	Mo *K*α radiation, λ = 0.71073 Å
Hall symbol: -P 2ybc	Cell parameters from 2622 reflections
*a* = 8.3747 (17) Å	θ = 3.1–27.5°
*b* = 13.772 (3) Å	µ = 2.30 mm^−^^1^
*c* = 12.153 (2) Å	*T* = 298 K
β = 93.89 (3)°	Needle, colourless
*V* = 1398.4 (5) Å^3^	0.36 × 0.32 × 0.28 mm
*Z* = 4	

### Data collection

**Table:**

Rigaku SCXmini diffractometer	3200 independent reflections
Radiation source: fine-focus sealed tube	2899 reflections with *I* > 2σ(*I*)
Graphite monochromator	*R*_int_ = 0.038
Detector resolution: 13.6612 pixels mm^-1^	θ_max_ = 27.5°, θ_min_ = 3.2°
ω scans	*h* = −10→10
Absorption correction: multi-scan (*CrystalClear*; Rigaku, 2005)	*k* = −17→17
*T*_min_ = 0.441, *T*_max_ = 0.525	*l* = −15→15
14246 measured reflections	

### Refinement

**Table:**

Refinement on *F*^2^	Secondary atom site location: difference Fourier map
Least-squares matrix: full	Hydrogen site location: inferred from neighbouring sites
*R*[*F*^2^ > 2σ(*F*^2^)] = 0.026	H atoms treated by a mixture of independent and constrained refinement
*wR*(*F*^2^) = 0.059	*w* = 1/[σ^2^(*F*_o_^2^) + (0.0208*P*)^2^ + 0.6019*P*] where *P* = (*F*_o_^2^ + 2*F*_c_^2^)/3
*S* = 1.15	(Δ/σ)_max_ < 0.001
3200 reflections	Δρ_max_ = 0.46 e Å^−^^3^
150 parameters	Δρ_min_ = −0.48 e Å^−^^3^
0 restraints	Extinction correction: *SHELXL97* (Sheldrick, 2008), Fc^*^=kFc[1+0.001xFc^2^λ^3^/sin(2θ)]^-1/4^
Primary atom site location: structure-invariant direct methods	Extinction coefficient: 0.0332 (7)

### Special details

**Table:**

Geometry. All e.s.d.'s (except the e.s.d. in the dihedral angle between two l.s. planes) are estimated using the full covariance matrix. The cell e.s.d.'s are taken into account individually in the estimation of e.s.d.'s in distances, angles and torsion angles; correlations between e.s.d.'s in cell parameters are only used when they are defined by crystal symmetry. An approximate (isotropic) treatment of cell e.s.d.'s is used for estimating e.s.d.'s involving l.s. planes.
Refinement. Refinement of *F*^2^ against ALL reflections. The weighted *R*-factor *wR* and goodness of fit *S* are based on *F*^2^, conventional *R*-factors *R* are based on *F*, with *F* set to zero for negative *F*^2^. The threshold expression of *F*^2^ > σ(*F*^2^) is used only for calculating *R*-factors(gt) *etc*. and is not relevant to the choice of reflections for refinement. *R*-factors based on *F*^2^ are statistically about twice as large as those based on *F*, and *R*- factors based on ALL data will be even larger.

### Fractional atomic coordinates and isotropic or equivalent isotropic displacement parameters (Å^2^)

**Table:**

	*x*	*y*	*z*	*U*_iso_*/*U*_eq_	
Cd1	0.77481 (2)	0.228314 (13)	0.006713 (15)	0.02939 (9)	
Cl2	0.78074 (8)	0.24012 (5)	0.21119 (5)	0.03509 (16)	
Cl3	0.80985 (8)	0.40133 (4)	−0.03853 (5)	0.03183 (15)	
Cl4	0.51643 (8)	0.15674 (5)	−0.05148 (5)	0.03630 (16)	
Cl1	1.01183 (8)	0.13969 (5)	−0.04509 (6)	0.03678 (16)	
N2	0.3731 (2)	0.42550 (13)	0.76461 (15)	0.0207 (4)	
C4	0.1866 (3)	0.28852 (19)	0.7311 (2)	0.0314 (6)	
H4A	0.1242	0.2852	0.6610	0.038*	
H4B	0.1932	0.2238	0.7625	0.038*	
N3	0.1084 (2)	0.35561 (15)	0.80727 (17)	0.0259 (4)	
C2	0.2107 (3)	0.36440 (19)	0.9119 (2)	0.0286 (5)	
H2A	0.2340	0.3005	0.9425	0.034*	
H2B	0.1554	0.4016	0.9654	0.034*	
C8	0.5761 (3)	0.55142 (19)	0.8006 (2)	0.0318 (6)	
C3	0.3525 (3)	0.32615 (19)	0.7140 (2)	0.0350 (6)	
H3A	0.4319	0.2822	0.7479	0.042*	
H3B	0.3681	0.3295	0.6358	0.042*	
C7	0.5335 (3)	0.46373 (17)	0.7366 (2)	0.0279 (5)	
H7A	0.6144	0.4143	0.7522	0.033*	
H7B	0.5307	0.4787	0.6585	0.033*	
C1	0.3652 (3)	0.4152 (2)	0.88691 (19)	0.0305 (5)	
H1A	0.3695	0.4788	0.9212	0.037*	
H1B	0.4561	0.3778	0.9170	0.037*	
N1	0.6122 (3)	0.61640 (17)	0.8522 (2)	0.0438 (6)	
C6	0.0823 (3)	0.45326 (19)	0.7556 (2)	0.0359 (6)	
H6A	0.0400	0.4978	0.8081	0.043*	
H6B	0.0057	0.4484	0.6922	0.043*	
C5	0.2410 (3)	0.4904 (2)	0.7198 (3)	0.0401 (7)	
H5A	0.2391	0.4918	0.6399	0.048*	
H5B	0.2592	0.5560	0.7469	0.048*	
H1	0.024 (4)	0.333 (2)	0.817 (3)	0.050 (10)*	

### Atomic displacement parameters (Å^2^)

**Table:**

	*U*^11^	*U*^22^	*U*^33^	*U*^12^	*U*^13^	*U*^23^
Cd1	0.02778 (12)	0.02888 (12)	0.03141 (13)	−0.00082 (7)	0.00131 (8)	0.00145 (7)
Cl2	0.0308 (3)	0.0461 (4)	0.0282 (3)	0.0020 (3)	0.0009 (3)	0.0045 (3)
Cl3	0.0360 (3)	0.0254 (3)	0.0348 (3)	0.0025 (2)	0.0080 (3)	−0.0005 (2)
Cl4	0.0314 (3)	0.0443 (4)	0.0334 (3)	−0.0076 (3)	0.0032 (3)	−0.0087 (3)
Cl1	0.0341 (3)	0.0352 (3)	0.0414 (4)	0.0051 (3)	0.0048 (3)	−0.0012 (3)
N2	0.0200 (9)	0.0205 (9)	0.0216 (9)	0.0004 (7)	0.0024 (7)	−0.0003 (7)
C4	0.0300 (13)	0.0308 (13)	0.0338 (14)	−0.0037 (10)	0.0046 (11)	−0.0111 (10)
N3	0.0195 (10)	0.0303 (11)	0.0283 (11)	−0.0023 (8)	0.0044 (8)	−0.0023 (8)
C2	0.0292 (13)	0.0334 (13)	0.0234 (12)	−0.0054 (10)	0.0017 (10)	−0.0007 (10)
C8	0.0288 (13)	0.0309 (14)	0.0351 (14)	−0.0056 (10)	−0.0013 (11)	0.0092 (11)
C3	0.0356 (14)	0.0293 (13)	0.0415 (15)	−0.0075 (11)	0.0140 (12)	−0.0176 (11)
C7	0.0250 (12)	0.0286 (12)	0.0306 (13)	−0.0040 (10)	0.0063 (10)	0.0026 (10)
C1	0.0272 (13)	0.0440 (15)	0.0203 (12)	−0.0044 (11)	0.0024 (10)	−0.0012 (10)
N1	0.0557 (16)	0.0299 (12)	0.0442 (14)	−0.0120 (11)	−0.0080 (12)	0.0085 (11)
C6	0.0258 (13)	0.0368 (14)	0.0449 (16)	0.0084 (11)	0.0017 (11)	0.0057 (12)
C5	0.0296 (13)	0.0315 (14)	0.0578 (18)	0.0028 (11)	−0.0060 (12)	0.0187 (13)

### Geometric parameters (Å, º)

**Table:**

Cd1—Cl4	2.4385 (8)	C2—H2A	0.9700
Cd1—Cl1	2.4486 (8)	C2—H2B	0.9700
Cd1—Cl3	2.4674 (8)	C8—N1	1.123 (3)
Cd1—Cl2	2.4874 (8)	C8—C7	1.467 (3)
N2—C5	1.496 (3)	C3—H3A	0.9700
N2—C1	1.499 (3)	C3—H3B	0.9700
N2—C7	1.503 (3)	C7—H7A	0.9700
N2—C3	1.505 (3)	C7—H7B	0.9700
C4—N3	1.491 (3)	C1—H1A	0.9700
C4—C3	1.511 (4)	C1—H1B	0.9700
C4—H4A	0.9700	C6—C5	1.515 (4)
C4—H4B	0.9700	C6—H6A	0.9700
N3—C2	1.489 (3)	C6—H6B	0.9700
N3—C6	1.494 (3)	C5—H5A	0.9700
N3—H1	0.79 (3)	C5—H5B	0.9700
C2—C1	1.520 (3)		
			
Cl4—Cd1—Cl1	116.28 (3)	N2—C3—C4	109.66 (19)
Cl4—Cd1—Cl3	116.26 (3)	N2—C3—H3A	109.7
Cl1—Cd1—Cl3	108.26 (3)	C4—C3—H3A	109.7
Cl4—Cd1—Cl2	105.86 (3)	N2—C3—H3B	109.7
Cl1—Cd1—Cl2	109.14 (3)	C4—C3—H3B	109.7
Cl3—Cd1—Cl2	99.49 (2)	H3A—C3—H3B	108.2
C5—N2—C1	109.6 (2)	C8—C7—N2	110.9 (2)
C5—N2—C7	111.00 (18)	C8—C7—H7A	109.5
C1—N2—C7	110.95 (18)	N2—C7—H7A	109.5
C5—N2—C3	109.5 (2)	C8—C7—H7B	109.5
C1—N2—C3	107.91 (19)	N2—C7—H7B	109.5
C7—N2—C3	107.77 (18)	H7A—C7—H7B	108.0
N3—C4—C3	108.67 (19)	N2—C1—C2	109.70 (19)
N3—C4—H4A	110.0	N2—C1—H1A	109.7
C3—C4—H4A	110.0	C2—C1—H1A	109.7
N3—C4—H4B	110.0	N2—C1—H1B	109.7
C3—C4—H4B	110.0	C2—C1—H1B	109.7
H4A—C4—H4B	108.3	H1A—C1—H1B	108.2
C2—N3—C4	109.2 (2)	N3—C6—C5	108.6 (2)
C2—N3—C6	110.2 (2)	N3—C6—H6A	110.0
C4—N3—C6	110.8 (2)	C5—C6—H6A	110.0
C2—N3—H1	112 (2)	N3—C6—H6B	110.0
C4—N3—H1	107 (2)	C5—C6—H6B	110.0
C6—N3—H1	108 (2)	H6A—C6—H6B	108.4
N3—C2—C1	108.38 (19)	N2—C5—C6	109.6 (2)
N3—C2—H2A	110.0	N2—C5—H5A	109.8
C1—C2—H2A	110.0	C6—C5—H5A	109.8
N3—C2—H2B	110.0	N2—C5—H5B	109.8
C1—C2—H2B	110.0	C6—C5—H5B	109.8
H2A—C2—H2B	108.4	H5A—C5—H5B	108.2
N1—C8—C7	177.4 (3)		

### Hydrogen-bond geometry (Å, º)

**Table:**

*D*—H···*A*	*D*—H	H···*A*	*D*···*A*	*D*—H···*A*
N3—H1···Cl2^i^	0.79 (3)	2.54 (3)	3.193 (2)	141 (3)
N3—H1···Cl3^ii^	0.79 (3)	2.76 (3)	3.285 (2)	126 (3)
C1—H1*A*···Cl3^iii^	0.97	2.70	3.507 (3)	141 (2)
C3—H3*B*···Cl4^iv^	0.97	2.67	3.599 (3)	160 (2)
C4—H4*A*···Cl1^i^	0.97	2.81	3.704 (3)	153 (2)
C7—H7*A*···Cl2^iv^	0.97	2.61	3.514 (3)	155 (2)
C7—H7*B*···Cl4^v^	0.97	2.79	3.489 (3)	129 (2)

Symmetry codes: (i) *x*−1, −*y*+1/2, *z*+1/2; (ii) *x*−1, *y*, *z*+1; (iii) −*x*+1, −*y*+1, −*z*+1; (iv) *x*, −*y*+1/2, *z*+1/2; (v) −*x*+1, *y*+1/2, −*z*+1/2.
